# The significance of managers’ knowledge of common mental disorders on managerial stigma towards employee depression: a cross-sectional study in Sweden

**DOI:** 10.1186/s12889-023-17577-5

**Published:** 2024-02-05

**Authors:** Carin Staland-Nyman, Kazi Mohammad Nurul Basar, Jenny Hultqvist, Monica Bertilsson

**Affiliations:** 1 School of Public Health and Community Medicine, Sahlgrenska Academy, University of Gothenburg, Gothenburg, Box 100, S-405 30 Sweden; 2 School of Health and Welfare, Halmstad University, Box 823, S-301 18 Halmstad, Sweden; 3 Institute of Neuroscience and Physiology, Sahlgrenska Academy, University of Gothenburg, Box 100, S-405 30 Gothenburg, Sweden

**Keywords:** Common mental disorders (CMD), Working-age population, Managers, Employees, Attitudes, Managerial stigma, Knowledge and understanding, Experience-based knowledge

## Abstract

**Background:**

Common mental disorders (CMD) such as depression, anxiety and stress-related disorders have increased in the working-age population in many countries but are still often associated with social stigma in workplaces. Managers have a key role in supporting employees with impaired health. Identifying factors that can improve stigmatizing attitudes among managers towards CMD is crucial. The aim of this study was to investigate managers’ knowledge of CMD on managerial stigma; more specifically knowledge aquired through training and education and through occupational and personal experience of CMD on low managerial stigma towards employee depression.

**Methods:**

Data from a web-based survey conducted in 2017 among 3038 managers in Sweden were used. Managers’ attitudes towards employee depression were measured using the Swedish version of the Managerial Stigma towards Employee Depression questionnaire. Binary logistic regression analysis, with adjustments for work setting and managerial experience, was conducted for associations between sources of knowledge of CMD and low managerial stigma.

**Results:**

With regard to knowledge acquired through training, medical training on CMD was significantly associated with a higher probability for low managerial stigma towards employee depression after adjustments (odds ratio [OR], 1.95; 95% confidence interval [CI], 1.26–3.01), whereas no significant associations were found between knowledge acquired through managerial training on CMD or level of formal education and low managerial stigma. With regard to knowledge acquired through professional and personal experience, occupational experience of treating people with CMD was significantly associated with a higher probability for low managerial stigma (OR, 2.03; 95% CI, 1.40–2.94) as was occupational experience of employees with CMD (1 employee: OR, 1.31; 95% CI, 1.04–1.66); >1 employee, OR 1.35 (CI 1.05–1.73). Personal experience of CMD was significantly associated with low managerial stigma (OR, 1.98; 95% CI, 1.60–2.46).

**Conclusions:**

Managers’ knowledge and understanding of CMD may increase the probability of a low level of managerial stigma towards employees with depression. Managers’ professional and/or personal experiences of CMD were important sources of knowledge in relation to a low level of stigmatizing attitudes. Organizations should encourage the use of managers’ experience-based knowledge of CMD in addition to training on CMD to reduce managerial stigma.

## Background

The prevalence of common mental disorders (CMD) (i.e. depression, anxiety, and stress-related disorders) has increased in many countries worldwide and is showing few signs of abating. More than one in six individuals in the European Union have been reported to have had a mental health disorder. Depressive, anxiety and stress-related disorders account for the highest prevalences [1]. Loss of health due to mental illness is substantial in the working-age population, and 80% of the global burden of mental health disorders occurs in people aged between 16 and 65 years [2]. CMD are among the most common and most costly health problems affecting the working-age population across ages, occupations, and industries [3, 4]. From a public health perspective, this is important because loss of societal and economic participation has negative consequences for employees, employers and societies.

It is known that CMD may adversely affect an individual’s capacity to work and may diminish work performance [5, 6]. Reduced capacity for motivation, learning, executive functioning, and commitment to work tasks or reduced capacity to interact with clients or colleagues in an adaptive manner due to depression or anxiety will likely adversely affect the work output [7, 8]. From an employer perspective, CMD may imply invisible costs due to employees’ reduced work performance while at work, and workplace costs have been identified as accounting for the largest portion of the increasing economic burden due to CMD [4]. Regarding depression, even a lower level of illness has been shown to be associated with decreased work performance, although the impact worsens as the severity increase [9, 10]. Illness severity also affects the probability of work absenteeism. Previous studies have shown that CMD increase the risk of sickness absence [11–14] and increase the risk of recurrences [15, 16].

Disabilities from CMD have also been shown to lead to long-term public health consequences with increased probability of temporary or permanent exclusion from work life through unemployment, disability pension and early retirement [17–21]. A study on a working population aged 20–35 years, with a 6-year follow-up, showed an eight times higher risk of disability pension and 29% higher risk of long-term unemployment in individuals with affective disorders (i.e. depression and anxiety) after adjustment for background factors, including somatic disorder and previous labour market attachment, than individuals without mental disorders [22]. Considering the increase in CMD in the working population and the far-reaching consequences for employees and workplaces (and for societies), there is a strong case for employers to strengthen knowledge on how to manage and prevent disability outcomes due to CMD. European employers have a constitutional duty to monitor and manage risk factors in the work environment in accordance with the European Directive 89/391/EEC-OSH [23]. For employers in Sweden, where this study was conducted, the Swedish Working Environment Act [24], further states that employers are required to adapt the working environment to their employees’ health conditions.

Based on employer responsibilities and the view of the workplace as a potential arena for preventing and counteracting aggravated health problems, the role of managers has been highlighted [25–27]. Among other factors, managers’ knowledge and attitudes regarding mental disorders and their influence on the prevailing workplace culture surrounding mental health have been discussed [28–30]. The need for increased knowledge on mental health among managers has also been emphasized by the World Health Organization (WHO) in their strategies concerning mental health at work, including interventions to prevent, protect and support employees with mental health conditions [26].

Managers have a pivotal role in being cognizant of health concerns among their employees and in offering support to employees affected by CMD [31–33]. Most employees spend much of their days at work, and managers (and colleagues) may be the first to notice symptoms of a CMD at an early stage. Furthermore, managers are well positioned to influence their employees’ pathways at work in times of health problems by being familiar with employee work requirements, having the authority to review and adjust work assignments and to identify preventive or supportive work accommodations when needed [34, 35]. However, negative and stigmatizing attitudes towards CMD may adversely affect managers’ motivation to consider supportive actions for employees with CMD. Recent research has found managers with stigmatizing attitudes to depression are less likely to take action at work to prevent CMD [36]. Stigma is still a major issue in public health, and it is well known that mental health disorders are often accompanied by social stigma whereby stereotypes and prejudices may lead to discriminatory behaviours [37, 38]. Moreover, social stigma has been found to be common within work settings and create barriers in the workplace for employees with mental disorders [29, 39]. Few studies have investigated the factors that may be related to less or non-stigmatizing attitudes in managers towards employees with CMD. In a previous study [30], managers in the public sector were found to have a less negative attitude towards employee depression compared with their counterparts in the private sector. Significant differences were also found in that study in relation to education and gender; educated managers versus less educated managers, and female managers versus male managers were found to have less negative attitudes towards employee depression. In a recent study from Sweden investigating gender differences with regard to attitudes towards employee depression among managers, female managers were found to have less negative attitudes than their male counterparts [40]. In addition, results from interventional studies have shown promising results on less negative attitudes in relation to managers’ participation in training sessions aimed at improving mental health awareness [41, 42].

The paucity of research investigating what factors and mechanisms are associated with low managerial stigma has been highlighted [43]. To date, the importance of different sources of knowledge that might be involved in improvement of managers’ attitudes to employee depression is understudied and has not been investigated among managers in a Swedish work context. To the best of our knowledge, no previous large study representing managers from different work sectors and managerial positions has investigated the influence of knowledge of CMD from different sources on low managerial stigma. Expressed in another way: are different sources of knowledge differently associated with a low level of stigmatizing attitudes. The stigma surrounding mental disorders at work, including managerial stigma, remains a major barrier to implementation of support at work for people living and working with a CMD. Investigating factors that may lead to less stigmatizing attitudes to CMD among managers is therefore an important step in capacity building and is in line with the strategies on mental health at work expressed by the WHO [26].

### Aim

The main objective of this study was to investigate managers’ knowledge of CMD and low managerial stigma; more specifically, to investigate the impact of managers’ knowledge of CMD acquired through training and education and through professional and personal experience on low level of stigmatizing attitudes towards employee depression.

## Methods

### Study design

The study had a cross-sectional design based on survey data from Sweden collected in 2017. The study was part of the project, Managers’ Perspective– A Missing Piece, with an overall aim to investigate managers’ attitudes and knowledge on CMD and to improve managers’ strategies and preventive measures in supporting employees with a CMD [31, 40].

Participants were recruited to the project from two sources: the Laboratory of Opinion Research (LORE) at the University of Gothenburg, Sweden, and the HELIX Competence Centre at the University of Linköping, Sweden. LORE (https://lore.gu.se/surveys/citizen/aboutcp) is a citizen panel used for various research purposes. The HELIX Centre (https://liu.se/en/research/helix-competence-centre) is a collaborative centre for 22 public and private organizations aiming to promote sustainable development in organizations.

Recruitment was conducted in several steps. As a first step, eligible participants for the project were identified through questions on managerial position included in the LORE 26th panel survey in 2017 [44]. Among the eligible participants identified, 5000 managers (age 20–65 years) were randomly selected. Another 556 eligible participants were identified from the HELIX Competence Centre. In a second step, the managers identified from the two recruitment sources were invited to participate in the project.

### Study population

In total, 5556 individuals were invited to participate in the project. Some individuals were excluded (n = 24) because of invalid e-mail information or other technical errors. Those individuals who did not hold a managerial position at the time of the web survey (n = 795) were also excluded. In total, 4737 eligible participants were identified.

External dropout (1379 individuals) was due to non-response (n = 963) and actively declining participation (n = 416). The distribution of dropouts from the two recruitment sources was 70% from the LORE sample and 30% from the HELIX sample. The gender distribution in the LORE sample showed a difference in proportion between the participants and the non-responders. Among men, the figures were 69% compared to 75%. For women the result was inversed, with 31% among the participants and 24% among the non-responders. No significant difference was found between participants and non-responders in the younger age-group, 20–49 years. However, there was a difference in the older age group, 50–59 years, with 36% among participants and 30%, among the non-responders. Regarding education, no significant difference was found between participants and non-responders. The study population included a higher proportion of managers in the public sector than in the private sector compared to the distribution at the Swedish labor market in general. The difference in distribution was 41% versus 21% for the public sector and, 59% versus 79%, for the private sector [45].

**Fig. 1:**
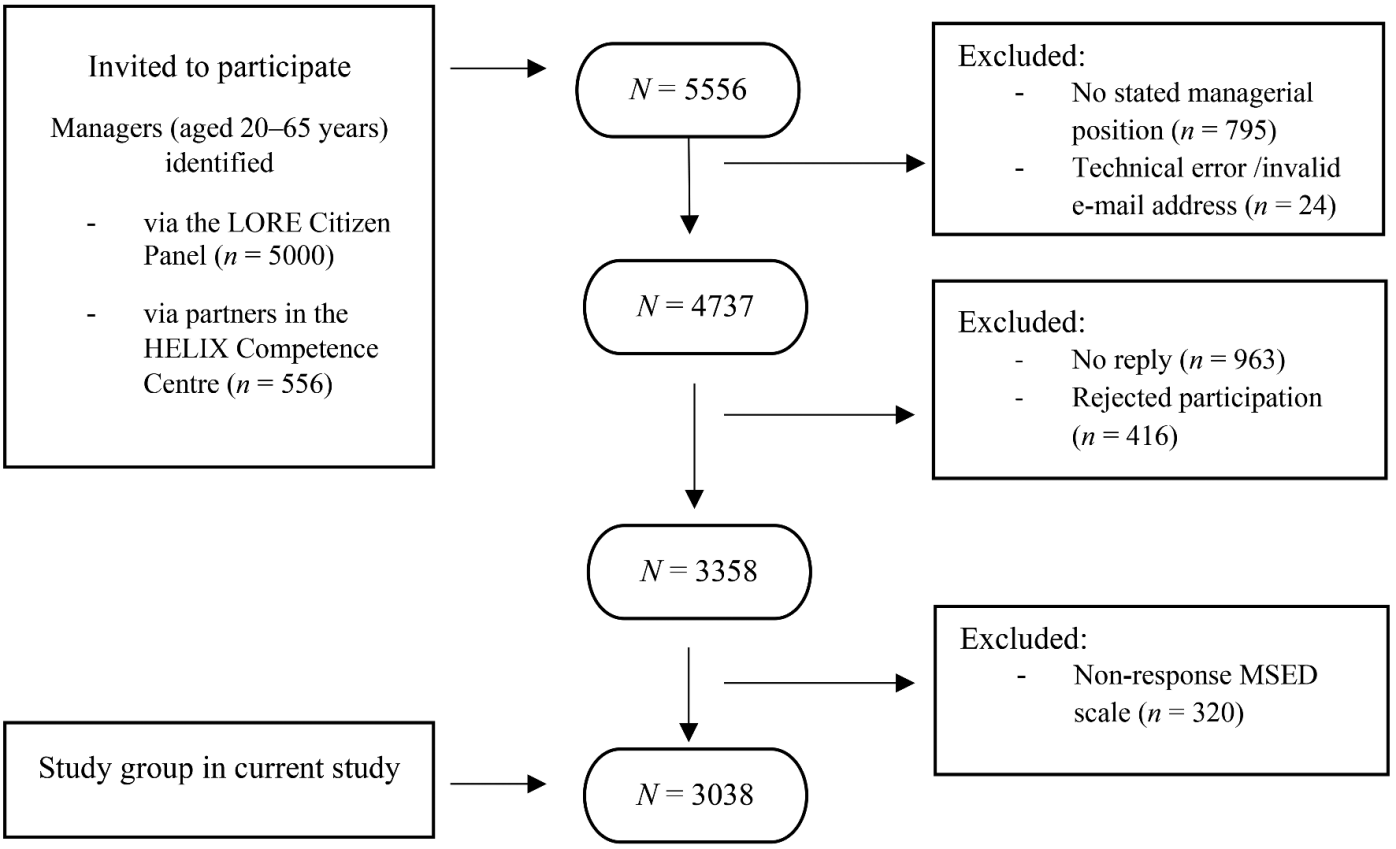
Flow chart of the study population in the earlier project, Managers’ Perspective– The missing piece 2017, and the study group in the current study. MSED, Managerial stigma towards employee depression scale [40]

Internal drop-out in certain questions was identified using the AAPOR standard definition for respondents [46] among the total respondents and showed that 84% of the respondents answered 80% or more of the questions. Because the study aim was to investigate determinants that could influence managerial stigma towards employees with depression, participants who did not answer all items in the Managerial Stigma towards Employee Depression (MSED) instrument were excluded (n = 320). Thus, the final study group in the current study comprised 3038 managers (flowchart presented in Fig. 1). The characteristics of the study group are presented in Table 1.

**Table 1:** Characteristics of the study group (*N* = 3038)

	Number	%
**Independent variables**		
*Knowledge aquired through education and training*		
Level of formal education		
Low and medium education^1^	1085	35.7
High education^2^	1945	64.0
Missing values	8	0.3
Medical training on CMDs		
Yes	343	11.3
No	2461	81.0
Missing values	234	7.7
Management training on CMDs		
Yes	744	24.5
No	2060	67.8
Missing values	234	7.7
*Knowledge through professional and personal experience on CMD*		
Occupational experience of employees with CMDs		
Yes, > 1 subordinate	875	28.8
Yes, 1 subordinate	911	30.0
No or do not know	1013	33.3
Missing values	239	7.9
Occupational experience of treating people with CMDs		
Yes	450	16.1
No	2352	83.9
Missing values	236	7.8
Personal experience of CMDs		
Yes	2148	70.7
No	657	21.6
Missing values	233	7.7
**Covariates**		
*Person-related characteristics*		
Gender		
Male	2030	66.8
Female	995	32.8
Non-binary	3	0.1
Missing values	10	0.3
Managerial position		
Senior or operations managers	907	29.9
Middle/first-line managers and supervisors	2054	67.6
Missing values	77	2.5
Managerial experience		
0–5 years	973	28.7
>5 years	2131	70.1
Missing values	34	1.1
Organization-related characteristics		
Work sector		
Private	1777	58.5
Public or non-profit	1259	41.4
Missing values	2	0.1
Industry^3^		
Blue collar	937	30,8
White collar	691	22,7
Pink collar	933	30,7
Other type	472	15,5
Missing values	5	0.3
Number of staff		
0–250	1530	50.4
>250	1505	49.5
Missing values	3	0.1
Staff gender composition		
More women	1039	34.2
As many women as men	836	27.5
More men	1152	37.9
Missing values	11	0.4

CMD, common mental disorder

^1^Compulsory school, upper secondary school, or equivalent post-secondary education

^2^University or university college

^3^Categorization of industries as shown in Table 2

### Data collection

Data were collected through a web-based survey in 2017. The survey, with two reminders, was distributed by LORE. The questionnaire comprised questions on managers’ knowledge, experiences, and strategies in relation to employees with reduced capacity to work due to depression or anxiety. Because the concept of CMD was not judged to be familiar to a broader population, the words “depression” and “anxiety,” were used throughout the questionnaire as proxy for CMD. Information about the background and the aim of the project was sent by e-mail to the invited participants. All participants were asked to provide informed consent. The study was approved by the Regional Ethical Review Board at Gothenburg University, Sweden (registration number: 165 − 17).

### Measurements

#### Exposure variables

The overarching factor of exposure in this study was managers’ knowledge about CMD. Knowledge can be acquired from different sources. In this study, managers’ self-reported knowledge was measured as knowledge through medical and managerial training on CMD (i.e., depression and anxiety) and knowledge through professional and personal experience of CMD. In addition, general knowledge through education, in terms of level of formal education, was measured. The reason behind this was, that even though formal education per se does not entail knowledge of CMD, the level of education has been shown to be of importance in mental health stigma [47].

Knowledge through medical and management training on CMD was measured by the two following questions: “Do you have any medical training that provides you with knowledge about depression and/or anxiety disorders?” with yes/no response options. “Have you participated in any managerial training where you received information about what you as a manager can do to support a staff member with depression and/or anxiety?” with three response options: “yes, during the last two years”, “yes, more than two years ago” or “no”. The responses were dichotomized into yes and no for the analyses.

Knowledge through professional and personal experience of CMD was measured by three questions covering experiences from a managerial position, a professional position and personal position. “During the past two years, have you had staff members at your current workplace who have had depression and/or anxiety disorders?” with the following response options: “yes, several staff members”, “yes, one staff member”, “no, no staff member” and “I don’t know”. The responses were trichotomized into yes < 1 staff member, yes > 1 staff member and no (this category also included responses that stated, I don’t know) for the analyses. “During your professional life, have you worked in occupations where you cared for or treated people with depression and/or anxiety disorders?” Personal experience, was assessed with the question: “Have you personally, or a close relative or a friend, had depression and/or anxiety disorders?” Response options for questions covering professional and personal experiences were yes and no.

General knowledge through education, i.e., level of formal education, was measured by the question: “What is your highest completed level of formal education”, with four response options: “compulsory school”, “upper secondary school or equivalent”, “degree from college/university (minimum 3 years)”, or “other post-secondary education”. The responses were dichotomized into “low/medium education” and “high education” for the analyses.

#### Outcome variable

The outcome in this study was managers’ attitudes to employee depression. Managers’ self-reported attitudes were measured by the specific instrument, MSED. MSED was developed by Martin and colleagues with the aim of addressing potential stigma among managers on depression in employees [30, 48]. MSED addresses stigma and its potential stereotypes, prejudices, and discrimination described previously by Corrigan and colleagues [37] and measures a tricomponent model of managers’ attitudes (affective, cognitive, and behavioural attitudes) to employees with depression [48]. The MSED instrument encompasses 12 statements and a 6-point Likert scale (from 1, strongly disagree to 6, strongly agree) reflecting managers’ attitudes.

A Swedish version of the MSED instrument was developed for the project “Managers’ Perspective– A Missing Piece”. The MSED instrument was linguistically and culturally translated from the original English version into Swedish by professionals with linguistic and field knowledge. The Swedish version was pilot tested by nine managers representing different professional fields in Sweden. After adjustments, the Swedish version of the MSED instrument was back translated to English. In a final step, the Swedish version was reviewed by the developer of the original MSED instrument [48] and was considered conceptually and culturally equivalent to the original instrument. The average item inter-correlation of the Swedish version was checked with Cronbach’s alpha test and the internal consistency and reliability were found to be sufficient (α = 0.79) [49]. The MSED scores range between 12 and 72, with lower scores indicating a less negative attitude. For the analyses in the present study, MESD scores were dichotomized with a cut-off at the 3rd quartile into low (corresponding to scores between 12 and 35) and high (corresponding to scores ≥ 36) levels of stigmatizing attitudes [40].

#### Covariates

The relationship between managers’ knowledge on CMD and their attitudes to depression in employees may be influenced by individual and contextual correlates. The selection of covariates was based on previous scientific knowledge from studies on stigma and mental health and scientific knowledge from the current project on managers’ perspectives [30, 31]. The selected covariates were grouped into person-related characteristics and organization-related characteristics.

Person-related characteristics were measured by three questions: (1) “gender” (response options: women, men, and non-binary; non-binary responses were excluded from the analyses due to the small number [n = 3]); (2) “managerial position” (response options: senior manager, middle manager, first-line manager, group leader/supervisor, and expert/operations manager); response options were dichotomized into “senior and operations managers” and “middle/first-line managers and supervisors”; (3) “total years of managerial experience” response options: 0–2 years, 3–5 years, 6–10 years and > 10 years, dichotomized into 0–5 years and > 5 years for the analyses.

Organization-related characteristics were measured by four questions: (1) “work sector” (response options: “governmental”, “municipal”, “county council/ regional”, “private” and “non-profit organization/foundation”). Responses were dichotomized into private sector and public and non-profit sector (including all response options except, private) for the analyses. (2) “Industry” was measured according to the company’s/organization’s main activity, with 16 response options following the Swedish Standard Industrial Classification (SNI) 2007 [50]. Response options were categorized into four categories: blue collar industry (industries working with things), white collar industry (industries working with data), pink collar industry (industries working with people) and other type of industry, following the concept of Fine [51]. Categorization details are presented in Table 2. (3) “Number of staff” with the response options 0–9, 10–49, 50–250, 251–1000 and > 1000 employees. Responses were dichotomized into 0–250 employees and ≥ 251 employees for the analyses. The chosen cut-offs reflected the usual size of small, medium-sized and large enterprises and organizations in Sweden, respectively. (4) “Staff gender composition” with the response options “more women”, “as many women as men” and “more men”.

**Table 2:** Categorization of industries

Type of industry	Industries included
White collar	IT, information, and communications activities
	Financial and insurance activities
	Public administration and defence
	Legal, economic, scientific, and technological activities
Blue collar	Agriculture, forester, fishing
	Mineral extraction (industry)
	Manufacturing industry
	Construction and craftsmanship
	Provision of electricity, heat, water, sewage, waste
	Transport
Pink collar	Trade/commerce
	Hotel and restaurant operations
	Education
	Health care, social services
Other	Culture entertainment, recreation
	Other type of activity

Categorization followed the concept suggested by Fine [51]

### Statistics

Descriptive analyses were conducted for study group characteristics and included item and total scores for the MSED instrument. Gender differences in managerial stigma measured with the MSED instrument was investigated with t test for equality of means. Binary logistic regression analyses in five models were conducted yielding odds ratios (ORs) and their 95% confidence intervals (95% CIs) for associations between managers’ knowledge of CMD and their attitudes towards employee depression. To rule out strong inter-correlations between the selected independent variables, multicollinearity was checked using Spearman’s rho test. Bivariate logistic regression analysis was conducted for each independent variable to obtain crude ORs with 95% CIs for associations. Multivariate analysis was conducted adjusted by covariates entered separately (models 1– 4) in the following order: gender (model 1), work sector, industry, number of staff (model 2), staff gender composition (model 3), managerial position, total years of managerial work (model 4). In the final model, all covariates were entered simultaneously (model 5). All statistical analyses were conducted using IBM SPSS statistics 27 (IBM Corp., Armonk, NY).

## Results

About two-thirds of the managers had high formal education, but only a small proportion had received medical training on CMD (about 11%) or management training on CMD (about 24%). Slightly more than every other manager had experience of one or more employees with a CMD. A smaller proportion (16%) of the managers had experience of treating people with a CMD and more than two-thirds had experience of CMD through their own experience or through close friends or relatives with a CMD. A higher proportion of the participating managers were men (about 67%), middle/first-line managers or supervisors (about 68%) and had > 5 years in a managerial position. A higher proportion worked in the private sector and about two-thirds of the managers represented blue collar or pink collar industries. There were as many small as large workplaces (i.e., < 250 employees or > 250 employees) and an equal staff gender composition was reported by 28% of the managers (Table 1).

The distribution of managerial stigma using the MSED total sum score (dichotomized at the 3rd quartile) showed low managerial stigma for a predominant proportion of the managers. A gender difference (*P* < 0.001) was seen; 87.5% of the female managers had low managerial stigma versus 75.3% of their male counterparts. The proportion of high managerial stigma was 12.5% and 24.7% for female and male managers, respectively (Table 3).

**Table 3:** Managerial stigma in women and men dichotomized at the 3rd quartile of sum score in the study group¹

	Women (*n* = 995)		Men (*n* = 2030)	
	Number	%	Number	%
High level of managerial stigma	124	12.5	502	24.7
Low level of managerial stigma	871	87.5	1528	75.3

Managerial stigma (i.e. stigmatizing attitudes towards employee depression) was measured with the Swedish version of the Managerial Stigma towards Employee Depression instrument originally developed by Martin and Giallo [48]

¹*P* < 0.001 (two-tailed t-test)

### Association between managers’ knowledge of CMD and low managerial stigma towards employee depression

Significant associations were found between four of the sources of knowledge of CMD and low level of stigmatizing attitudes (i.e., low managerial stigma) (Table 4). Regarding knowledge acquired through training on CMD, only medical training on CMD was significantly associated with a higher probability for a low level of stigmatizing attitudes (OR, 1.95; 95% CI, 1.26–3.01) when all covariates were adjusted for simultaneously in the final model. No significant association was found between knowledge through managerial training on CMD and managerial stigma. Regarding knowledge through professional and personal experience, all sources of knowledge were significantly associated with low managerial stigma in the final model. Managers who had occupational experience of treating people with CMD had about twice as high probability for a low level of stigmatizing attitudes (OR, 2.03; 95% CI, 1.40–2.94) compared with managers lacking such experience. In relation to personal experience of CMD (i.e., through own experience or through a close friend or relative), the probability for a low level of stigmatizing attitudes was about twice as high (OR, 1.98; 95% CI, 1.60–2.46). Having occupational experience of employees with CMD was significantly associated with a low level of stigmatizing attitudes regardless of whether managers had previous experience of CMD in one employee (OR, 1.31; 95% CI, 1.04–1.66) or more than one employee (OR, 1.35; 95% CI, 1.05–1.73). Regarding formal level of education, the association with managerial stigma became non-significant when all covariates were adjusted for in the final model.

**Table 4:** Crude and adjusted odds ratios (ORs) with 95% confidence intervals (CIs) for managerial stigma towards employee depression comparing managers reporting low versus high levels of stigmatizing attitudes with respect to managers’ knowledge of CMD

Managers’ knowledge of CMDs	Number	OR (95% CI)					
		Crude	Model 1	Model 2	Model 3	Model 4	Model 5
**Knowledge of CMD acquired through training**							
Medical training on CMD							
No medical training on CMD	2453	1	1	1	1	1	1
Medical training on CMD	340	**2.70 (1.85–3.93)**	**2.50 (1.71–3.64)**	**1.86 (1.23–2.81)**	**2.40 (1.63–3.53)**	**2.64 (1.79–3.90)**	**1.95 (1.26–3.01)**
Management training on CMD							
No management training on CMD	2051	1	1	1	1	1	1
Management training on CMD	742	1.07 (0.87–1.32)	1.14 (0.92–1.41)	1.00 (0.81–1.25)	1.06 (0.86–1.31)	1.08 (0.87–1.34)	1.05 (0.83–1.31)
**Knowledge of CMD acquired through professional and personal experience**							
Occupational experience of employees with CMD							
No		1	1	1	1	1	1
Yes, 1	907	**1.55 (1.25–1.93)**	**1.46 (1.17–1.82)**	**1.42 (1.13–1.78)**	**1.47 (1.18–1.84)**	**1.44 (1.15–1.80)**	**1.31 (1.04–1.66)**
Yes, >1	871	**1.83 (1.45–2.30)**	**1.62 (1.29–2.05)**	**1.44 (1.13–1.83)**	**1.65 (1.31–2.08)**	**1.71 (1.35–2.17)**	**1.35 (1.05–1.73)**
Occupational experience of treating people with CMD							
No	2343	1	1	1	1	1	1
Yes	448	**2.59 (1.87–3.57)**	**2.38 (1.72–3.29)**	**1.93 (1.35–2.75)**	**2.27 (1.63–3.16)**	**2.58 (1.84–3.60)**	**2.03 (1.40–2.94)**
Personal experience of CMD^1^							
No	654	1	1	1	1	1	1
Yes	2140	**2.02 (1.65–2.47)**	**1.93 (1.57–2.36)**	**2.11 (1.71–2.60)**	**2.05 (1.67–2.51)**	**1.96 (1.59–2.41)**	**1.98 (1.60–2.46)**
**General knowledge aquired through education**							
Level of formal education							
Low and medium education^2^	1080	1	1	1	1	1	1
High education³	1938	**1.56 (1.30–1.86)**	**1.41 (1.18–1.70)**	1.18 (0.97–1.44)	**1.38 (1.15–1.66)**	**1.47 (1.22–1.77)**	1.10 (0.89–1.35)

Crude, bivariate analyses; Model 1, adjusted for gender; Model 2, adjusted for work sector, industry, staff numbers; Model 3, adjusted for staff gender composition; Model 4, adjusted for managerial position, years of managerial work experience; Model 5, adjusted for all covariates in Models 1–4. Significant results are shown in bold type.

CMD, common mental disorder

^1^Personal experience of CMDs includes own experience or through a close friend or relative

^2^Compulsory school, upper secondary school, or equivalent post-secondary education

^3^University or university college

## Discussion

The WHO states that stigma related to mental illness, including CMD, remains a barrier to the implementation of support at work or the uptake of existing support for people with mental illness [26]. Considering managers’ pivotal role in supporting employees with health conditions and their influence on the prevailing workplace culture surrounding mental health, factors promoting non-stigmatizing attitudes in managers have attracted interest [26, 27]. The main findings from the current study, encompassing a large number of managers representing various sectors and industries, support the fact that specific knowledge and understanding of CMD in managers may increase the probability of low managerial stigma towards employees with depression. The study also supports the importance of acknowledging managers’ professional and personal experiences of CMD as valuable sources of knowledge that can lead to low managerial stigma.

Specific knowledge in terms of medical education and training on mental illness has been shown previously to be associated with lower levels of stigmatizing attitudes in professionals [52]. This was confirmed in the current study in relation to managers. Managers with specific knowledge, such as medical training on CMD, were about twice as likely to report low managerial stigma towards employee depression compared with managers without such training. This was also true after adjustments for person-related and organization-related characteristics. About 50% of the managers with medical training, reported experience of having worked in occupations caring or treating people with depression/anxiety, which may have influenced the results on managerial stigma. The association between specific knowledge in terms of management training on CMD and low managerial stigma was non-significant. This was intriguing since earlier findings in this project showed that managers with management training on CMD seemed to be more prone to initiate preventive actions to support employees with depression [34]. However, in line with the result in the present study, findings from the project also showed that no association was established between management training on CMD and managers finding out about CMD among their employees [31].

The current study confirmed the significance of specific experience-based knowledge stemming from experiences of handling CMD in patients, employees or a relative, or personal illness. The impact of professional or personal experience of CMD on the probability for low managerial stigma was about the same as for medical training. In addition, minor occupational experience of employees with CMD among managers was associated with low managerial stigma. These findings correspond with research on public stigma on mental illness in which experience in terms of having personal contact with individuals with mental illness has been proven to be important in reducing prejudices and stigma concerning mental illness [47, 53]. In workplace interventions, a combination of approaches, e.g. using an educational approach together with facilitating personal contacts with people who have experienced mental illness, have been shown to reduce stigma [39]. The importance of experience-based knowledge on low managerial stigma is also in line with previous results showing that gaining more familiarity with how to handle different work-related situations that may occur in employees with a CMD has shown promising results in interventions on stigmatizing attitudes [41, 42, 54].

### Strengths and limitations

A strength of this study is the large study group of 3038 managers representing various work sectors and managerial positions. Moreover, the study group reflected the gender distribution among managers in Sweden and on the Swedish labour market [55]. The random selection process of participants minimized the possibility of selection bias, providing representativeness and increased generalizability. A further strength is the use of a valid and reliable instrument for measuring managers’ attitudes to depression in employees. The MSED instrument had undergone rigorous testing previously [48] and was considered suitable for a Swedish occupational context [40]. The comprehensive survey covering potential factors influencing the associations between managers’ knowledge and managerial stigma allowed an extensive confounding analysis on personal as well as contextual organization-related factors as suggested in previous studies [30, 56].

A limitation was the cross-sectional design, which did not allow for causal inference between different sources of knowledge and managerial stigma. Both exposure and outcome factors were self-reported and different types of bias such as social desirability or bias due to other reasons for self-assessments being under- or overestimated, cannot be ruled out. The results were generated in a national context where employers responsibility for employee health is enshrined in legal acts. Even though the responsibility rests with the employer, it may be possible due to managers’ pivotal role in the organization, that this affected reports on stigma. There was a significantly higher dropout rate among men than among women. Bearing in mind previous results [30] showing a higher prevalence of stigmatizing attitudes among men, the higher dropout rate among men may have affected the results in the present study. However, in contrast to previous results [30], there were no significant difference between the study population and the dropouts in relation to level of formal education. Regarding industry, a larger proportion of the managers in the study population worked in the public sector and a smaller proportion in the private sector, compared to the Swedish labour market in general. In line with many other web-based surveys, internal attrition was relatively high. However, 84% of the total respondents answered at least 80% of the questions in the survey.

### Implications and conclusions

Different sources of managers’ knowledge of CMD may be differently associated with managerial stigma. The importance of acknowledging managers’ professional or personal experience of CMD as important sources from which non-stigmatizing attitudes may develop was supported. In line with studies on workplace interventions aiming at reducing stigmatizing attitudes, the results from this study support the fact that knowledge and understanding may be based not only on education giving basic facts on CMD but also on experience and practice related to CMD. Knowledge from these different sources is integrally intertwined so that relevant and tailored knowledge applicable to work situations may be generated.

Considering the far-reaching consequences of CMD in the working-age population on an individual, organizational and societal level, knowledge of the factors that can be of significance in tackling stigmatizing attitudes towards CMD in workplaces and in managers is important. From this study, it can be suggested that to reduce potential prejudices against CMD, organizations should provide their managers with training and discussion on the perceptions and consequences of CMD in the specific work setting. Moreover, organizations need to increase their awareness, and strengthen their use of experience-based knowledge of CMD that may exist in managers in the organization. For example, organizations may consider managerial experience of supporting employees with CMD as one asset among other assets in a manager group. By emphasizing this knowledge being valued and demanded by the organization, this may contribute to improving attitudes towards non-managerial stigma.

Overall, the results from the present study show that specific knowledge and understanding in managers may help to dispel myths and misconceptions, which is central in tackling stigmatizing attitudes towards CMD in employees. Moreover, the results can contribute to capacity building in workplaces aiming at “preventing, protecting and promoting and supporting” mental health at work, which is in line with the WHO Guidelines on Mental Health at Work.

## Data Availability

The data used for this study are archived at LORE at the University of Gothenburg and can be obtained by contacting LORE at info@lore.gu.se or survey manager Maria Andreasson at maria.andreasson@gu.se.

## Abbreviations

CMD: common mental disorder
LORE: Laboratory of Opinion Research
MSED: Managerial Stigma towards Employee Depression
WHO: World Health Organization
